# Risk Comparison of the Diarrheal and Emetic Type of *Bacillus cereus* in Tofu

**DOI:** 10.3390/microorganisms7110536

**Published:** 2019-11-07

**Authors:** Mi Jin Kwon, Chae Lim Lee, Ki Sun Yoon

**Affiliations:** Department of Food and Nutrition, College of Human Ecology, Kyung Hee University, Seoul 02447, Korea; kmj9303@hanmail.net (M.J.K.); crl95@khu.ac.kr (C.L.L.)

**Keywords:** tofu, *Bacillus cereus*, diarrheal, emetic, biofilm, spore, survival, D-values

## Abstract

We investigated the ability of biofilm formation, survival, and behavior of diarrheal and emetic *Bacillus cereus* vegetative cells and spores in tofu. Both diarrheal and emetic *B. cereus* did not proliferate at a temperature below 9 °C in tofu. However, the emetic *B. cereus* grew faster than diarrheal *B. cereus* at 11 °C and had better survival ability at low temperatures. Both diarrheal and emetic *B. cereus* were able to form a biofilm on stainless steel. These biofilm cells were transferred to tofu in live state. The transferred biofilm cells could not grow at a temperature below 9 °C but grew over 11 °C, like planktonic cells. *B. cereus* contamination in tofu at a high concentration (>6 logs CFU/g) was not entirely killed by heating at 80, 85, or 90 °C for 2 h. Spores and emetic *B. cereus* had higher resistance to heat than vegetative cells and diarrheal *B. cereus*, respectively.

## 1. Introduction

Tofu, a soy-based food, is known to be healthy because it has low calories and large amounts of proteins (41.3%) with all nine essential amino acids. Also, it is an excellent source of iron, calcium, and other micro-nutrients [1]. Because of these health benefits, consumption of tofu has increased. Thus, the safety of tofu in the retail market should be guaranteed. Due to its high protein, high water content, and neutral pH, tofu also provides perfect conditions for the growth of microorganisms [2]. If proper manufacturing and storage procedures are not practiced, tofu may cause foodborne illness. Although types of bacterial contamination in raw materials or processing are different, numerous studies have shown that *Bacillus* spp. is a significant microbiological hazard in tofu [3]. Lee et al. [4] have also found two species of *B. cereus* in tofu stored at 37 °C for 24 h.

*B. cereus* can cause food spoilage and food poisoning by producing emetic and diarrheal toxins [5]. *B. cereus* produces endospores that are strongly resistant against wet or dry heat, radiation, desiccation, extreme pH, chemicals, enzymes, and high pressure [6]. Thus, its ability to form spores can be a potential hazard in heated food. When tofu is stored at an appropriate temperature, spores can germinate, regrow, and produce toxins that lead to foodborne disease [7]. Moreover, the microbial safety of tofu is continuously threatened by biofilm formation during the manufacturing process (personal communication). Biofilm cells can cause cross-contamination of foods, leading to short shelf life with quality deterioration or food poisoning [8]. Biofilm cells are also resistant to heat and can survive manufacturing and packaging processes.

To date, previous studies have focused on the identification of spoilage bacteria in tofu [9,10] or shelf-life extension of tofu [11,12]. Studies on behavioral characteristics of *B. cereus*, such as biofilm formation, cross-contamination, and regrowth potential in tofu, are lacking. Therefore, the objectives of this study are: (1) to compare growth and survival behaviors of diarrheal and emetic *B. cereus* in tofu at refrigerated and ambient temperatures, (2) to investigate the possibility of regrowth in tofu when biofilm is transferred to tofu, and (3) to compare the survival ability of diarrheal and emetic *B. cereus* in tofu during heat treatment.

## 2. Materials and Methods

### 2.1. Bacterial Strains

*Bacillus cereus* diarrheal (ATCC 11778) and emetic (NCCP 14796) strains were purchased from the Korean Federation of Culture Collections (KFCC) and stored frozen at −80 °C. Ten microliters of *B. cereus* was then inoculated into 10 mL of sterilized nutrient broth (NB; MB cell, Seoul, Korea). These inoculated bacteria were cultured at 36 °C for 24 h with shaking (140 rpm) using a rotary shaker (VS-8480, Vision, Daejeon, Korea) to reach a concentration of 8 log CFU/mL or more. Pre-cultured bacteria were diluted with sterilized 0.1% peptone water (BD Difco, Sparks, MD, USA).

### 2.2. Effect of Temperature on the Growth and Survival Characteristics of B. cereus in Tofu

The tofu was purchased from local supermarkets in Seoul, Korea. It was aseptically cut into 10 g (3 cm × 3 cm × 1 cm). Then 100 µL of pre-cultivated diarrheal or emetic *B. cereus* was inoculated into tofu, which was then placed in a 60 mm petri dish (SPL life Sciences, Pocheon, Korea) and stored at 4, 9, 10, 11, and 25 °C. The modified Gompertz equation [13] shown below was used as the growth model at each temperature.

Y = N0 + C × exp(−exp((2.718 × SGR/C) × (Lag−X) + 1))(1)

where N0 is the initial log number of cells, C is the difference between initial and final cell numbers, Lag is lag time (LT) before growth (h), SGR is a specific growth rate (log CFU/h), X is sampling time (h), and Y is log cell number (log CFU/g). LT and SGR values were calculated using Graph Pad Prism V4.0 (Graph-Pad Software, San Diego, CA, USA).

The modified Weibull equation shown below was used as the survival model at each temperature. Delta (δ) and p were calculated using GInaFiT V1.5 [14].

Log_10_(N) = log_10_(N0) − (t/delta)^p^ (2)

where N0 is the initial log number of cells, Delta (δ) is time for the first decimal reduction of a bacteria population, t is time (h), and p is the shape of the survival curve. The Weibull model corresponds to a concave upward curve if p < 1 and convex downward curve if p >1. The model is applicable for the frequently observed non-linear survival curves of microorganisms, and it can also describe first-order kinetics when p = 1.

### 2.3. Biofilm Formation

Stainless steel coupons (SUS 304, 2 cm × 2.5 cm) were cleaned in 70% alcohol to remove grease. Cleaned coupons were transferred to 50-mL conical tubes (SPL life Sciences, Pocheon, Korea) and sterilized in an autoclave before use [15]. Sterile coupons were vertically immersed in conical tubes containing 25 mL tryptic soy broth (TSB; MB cell, Seoul, Korea). Then 1 mL of pre-cultivated diarrheal or emetic strain was inoculated into conical tubes (approximately 10^6^ cells/mL) containing coupons [16]. These immersed coupons were incubated at 30 °C for 24 h without shaking. The coupons were then transferred to new conical tubes containing fresh medium and 1 mL of pre-cultivated *B. cereus.* Theses conical tubes were incubated at 30 °C for 24 h without shaking [17]. These bacteria were attached to both sides of coupons, which were then washed in sterile distilled water to remove loosely attached bacteria. Beads assay method [18] was used to confirm biofilm formation. Stainless steel coupons were placed in a sterile 50-mL conical tube containing 10 mL of sterile 0.1% peptone water and 10 glass beads (5-mm diameter; DAIHAN Scientific, Seoul, Korea) and vortexed for 2 min. The vortexed solution was serially diluted with 0.1% peptone water, spread onto tryptic soy agar (TSA; MB cell, Seoul, Korea), and incubated at 30 °C for 24 h. Results are expressed as log CFU per coupon (log CFU/coupon).

### 2.4. Transfer of Biofilm Cells on Stainless Steel Coupon to Tofu and Regrowth

The tofu was cut aseptically into 10 g (2.5 cm × 2 cm × 2 cm). Both sides of biofilm coupons were air-dried for 10 min at room temperature. The sandwich method [15,19] was used to transfer biofilm cells to tofu. Biofilm was formed on both sides of coupons, which were placed between 10 g of tofu pieces in a sandwich shape. Then 140 g of the tray was placed on the top of the tofu and left for 2.5 min to expose *B. cereus* biofilm cells to the tofu. This step was repeated with the bottom of the tofu in a sandwich shape. The pressing time was shifted up and down, with 2.5 min each for a total of 5 min.

The efficiency of transfer (EOT) was calculated using the equation described by Montville et al. [20].

(3) $$\mathrm{The}\;\mathrm{efficiency}\;\mathrm{of}\;\mathrm{transfer}\;(\mathrm{EOT})\;=\;\frac{\mathrm{CFU}\;\mathrm{on}\;\mathrm{destination}}{\mathrm{CFU}\;\mathrm{on}\;\mathrm{source}\;}$$

Biofilm-transferred tofu in a sandwich shape was then stored in sterile polypropylene containers (30-mL, DAIHAN Scientific, Seoul, Korea) at 4, 9, 11, and 25 °C. The possibility of regrowth of biofilm cells in tofu was examined. At a specific time, biofilm-transferred tofu was homogenized with sterilized 0.1% peptone water for 120 s using a stomacher. Then 1 mL aliquot of these homogenates was serially diluted with 0.1% peptone water, spread onto mannitol egg yolk polymyxin (MYP) agar, and incubated at 30 °C for 24 h. Results are expressed as log colony-forming units per gram (log CFU/g) of tofu.

### 2.5. Viability of the Transferred Biofilm Cells

*B. cereus* viability in biofilm cells was assayed using a LIVE/DEAD^®^ BacLightTM Bacterial Viability Kit (Invitrogen Inc., Eugene, OR, USA). One milliliter of biofilm cells obtained by beads assay was mixed with 3 µL of stain solution (SYTO^®^9 and Propidium iodide was mixed at the same ratio) and incubated at room temperature for 15 min in the dark. Dyed biofilm cells were observed under a fluorescence microscope (Nikon Eclipse E800; Nikon Instech Co., Ltd., Karagawa, Japan) equipped with TRITC and FITC filters (Nikon). Bacteria cells stained with SYTO^®^9 were green, representing live bacteria, while propidium iodide (PI)-stained bacteria cells were red, representing dead bacteria [21].

### 2.6. Spore Formation

Sporulation of *B. cereus* was made using nutrient agar (NA; MB cell, Seoul, Korea) supplemented with 1 ppm Mn^2+^. The developed nutrient agar was inoculated with 2 mL of pre-cultivated diarrheal or emetic *B. cereus* strains. Plates were then incubated at 30 °C for 7 days. Spores were harvested using a disposable, sterile plastic loop with 5 mL of cold, sterile McIlvaine buffer (pH 7.0), and centrifuged at 4,000× *g* for 20 min to remove vegetative cells and debris. This procedure was repeated three times. Pellets were resuspended with 10 mL of McIlvaine buffer, and spore solutions were stored at 0–5 °C until use [22].

### 2.7. B. cereus Survival during Heat Treatment

The tofu was cut aseptically into 10 g each (2.5 cm × 2 cm × 2 cm). Each piece of tofu was inoculated with 100 µL vegetable cells or spores of diarrheal or emetic strain. The inoculated tofu was placed in a sterile polypropylene container, and 20 mL of sterile distilled water was added to simulate actual heat-sterilization conditions of tofu during the manufacturing process. Heat treatment was carried out at 80, 85, and 90 °C in temperature-controlled water bath (EYELA, Tokyo, Japan). The polypropylene container containing the tofu inoculated with vegetable cells or spores was placed in a water bath, and survival ability of vegetable cells or spores was determined at appropriate time intervals. After heat treatment, samples were cooled in ice water (less than 0 °C). These cooled samples were homogenized with sterilized 0.1% peptone water for 120 s using a stomacher. Then 1 mL aliquot of homogenate was serially diluted with 0.1% peptone water, spread onto MYP agar, and incubated at 30 °C for 24 h. Results are expressed as log colony forming units per gram (log CFU/g) of tofu.

### 2.8. Calculation of D-values

D-values were calculated using the method described by Kim et al. [23]. Data at 80, 85, and 90 °C were used to plot a thermal death curve (TDC; log population of survivors against heating time). Linear fit for the plot was determined using linear regression in Graph Pad Prism V4.0 (Graph-Pad Software, San Diego, CA, USA). D-values were obtained by calculating the inverse negative of the slope at each temperature (D-value = −1/slope).

### 2.9. Statistical Analysis

All experiments were repeated twice with two replicates per treatment. T-test was performed to compare the behavioral characteristics of diarrheal and emetic *B. cereus*. Duncan’s multiple range test was performed to determine the significant difference in survival ability among *B. cereus* spores and vegetative cells. All statistical analyses were performed using statistical software program SAS, version 9.4 (SAS Institute, Inc., Cary, NC, USA). Statistical significance was set at *p* < 0.05.

## 3. Results and Discussion

### 3.1. Effect of Temperature on the Growth and Survival Characteristics of B. cereus Planktonic Cells in Tofu

**Figure 1:**
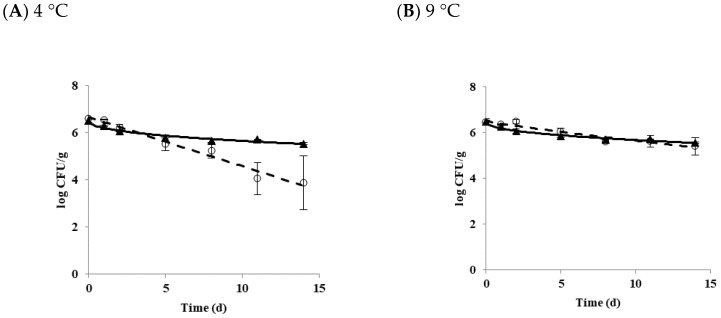

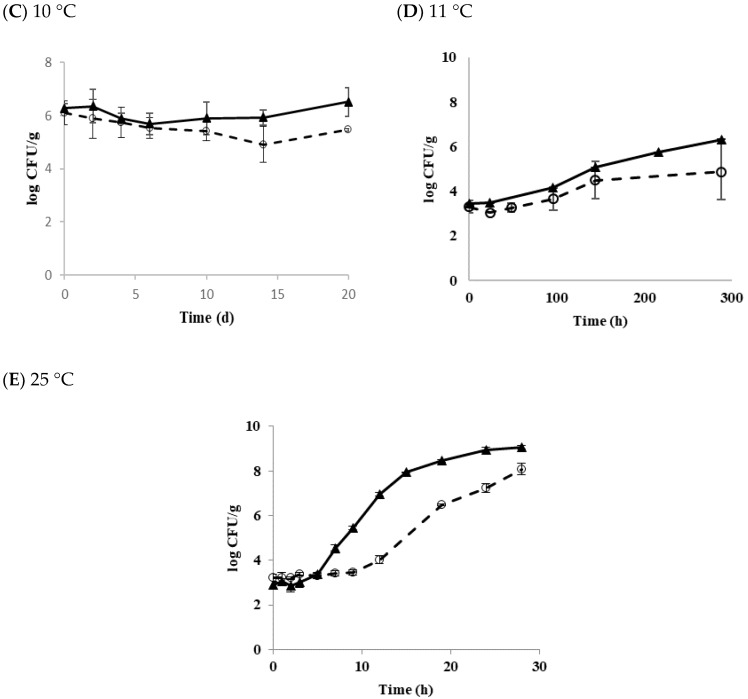
The growth and survival of *B. cereus* planktonic cells in tofu as a function of temperature. (**A**) 4 °C; (**B**) 9 °C; (**C**) 10 °C; (**D**) 11 °C; (**E**) 25 °C. ◯ Diarrheal strain; ▲ Emetic strain.

The growth and survival characteristics of *B. cereus* planktonic cells in tofu as a function of temperature is shown in Figure 1. The growth of *B. cereus* was not observed at 4 (Figure 1A) or 9 °C (Figure 1B). *B. cereus* did not grow or die at 10 °C (Figure 1C). Growth and survival kinetic parameters of *B. cereus* planktonic cells in tofu at 4, 9, 10, 11, and 25 °C are shown in Table 1. Delta (δ) values for diarrheal and emetic *B. cereus* in tofu stored at 4 °C were 5.15 and 14.0 days, respectively. When tofu was stored at 9 °C, delta values were 12.88 for the diarrheal strain and 22.59 days for the emetic strain. There were significant differences in delta values between these two types of strains (*p* < 0.05) at both 4 and 9 °C. *p* values, representing the shape of the graph, were less than 1 (concave curve) for both diarrheal and emetic *B. cereus* at all temperatures, indicating that microorganisms die rapidly at the beginning of storage [24].

Overall, the delta value at 9 °C (12.88 to 22.59) was higher than those at 4 °C (5.15 to 14.00), indicating that *B. cereus* decreased more rapidly at 4 °C, regardless of strain type. The lower the temperature, the faster the reduction of *B. cereus* was observed at the same time. When differences in delta values were compared between diarrheal strain and emetic strain, the delta values of the emetic strain (14.00 to 22.59) were higher than those of the diarrheal strain (5.15 to 12.88) while p values of the emetic strain were smaller (0.43 to 0.44) than those of the diarrheal strain (0.88 to 0.98) at 4 and 9 °C. These results indicated that the survival ability of emetic *B. cereus* strain was better than that of diarrheal *B. cereus* strain in tofu at both temperatures.

Results of the primary growth model of *B. cereus* at 11 and 25 °C are shown in Figure 1D,E, respectively. At 11 °C, LT value was 79.67 for diarrheal *B. cereus* and 45.39 h for emetic *B. cereus* in tofu, while the SGR value was equal to 0.02 log CFU/h for both strains. When tofu was stored at 25 °C, LT values of diarrheal and emetic strains were 9.53 and 4.34 h, and SGR values of diarrheal and emetic strains were 0.31 and 0.55 log CFU/h, respectively. There was a significant difference in LT and SGR values between these two types of strains (*p* < 0.05) at 25 °C. As shown in Table 1, emetic *B. cereus* grew faster than diarrheal *B. cereus* in tofu at temperatures above 11 °C. The distribution temperature of refrigerated foods in Korea is controlled at 0~10 °C [25]. The current guideline for refrigerated food is not appropriate for *B. cereus* contamination in tofu because 10 °C is the boundary temperature of growth and death of *B. cereus* in tofu, as shown in the present study. It has also been reported that *S. aureus* can grow at 8 °C [26], while the growth of *L. monocytogenes* was observed at 4 °C [27] in tofu. To prevent the growth of pathogenic bacteria in tofu at the retail market, careful monitoring of distribution temperature for refrigerated foods, including tofu, is needed.

### 3.2. Biofilm Formation and Transfer of Biofilm Cells

The ability of biofilm formation of *B. cereus* on stainless steel was compared between diarrheal and emetic *B. cereus*. The mean values of diarrheal and emetic biofilm cells on stainless steel were 4.01 ± 0.51 and 4.00 ± 0.71 log CFU/coupon, respectively (Table 2). The level of biofilm formation showed no significant difference between diarrheal and emetic *B. cereus* (*p* > 0.05). Biofilm formation is affected by various factors. Strains isolated from soil or strains involved in digestive tract infection are excellent biofilm formers, whereas strains isolated from other diseases are weak biofilm formers [28]. Also, pH [29], nutrients level [30], and temperature [31] play a role in the rate of microbial adherence to a substratum. The result of the present study showed that *B. cereus* could form biofilm on the food-contact surface, regardless of the type of strain. If *B. cereus* strains are not entirely removed from food-contact surfaces, they can grow to mature biofilm. Therefore, effective washing is essential to prevent and control biofilm formation in a tofu manufacturing environment.

Table 2 also shows the efficiency of transfer (EOT) of biofilm cells from stainless-steel to tofu. Although the EOT value of emetic *B. cereus* (0.81 ± 0.12) was higher than that of diarrheal *B. cereus* (0.72 ± 0.05), there was no significant difference in EOT value between these two types of strains (*p* > 0.05). Microorganisms contaminated onto the food-contact surface might be transferred to food. The extent of transfer depends on bacterial density on the surface, microbial adhesion strength to the surface, surface moisture, and moisture content of food [32,33]. Various *Bacillus* strains have been detected in equipment such as drainers and cutters in a tofu manufacturing environment [9]. This result demonstrates the possibility of biofilm formation of *B. cereus* on the food-contact surface and transfer to tofu during the manufacturing process. 

### 3.3. Viability of Transferred Biofilm Cells

**Figure 2:**
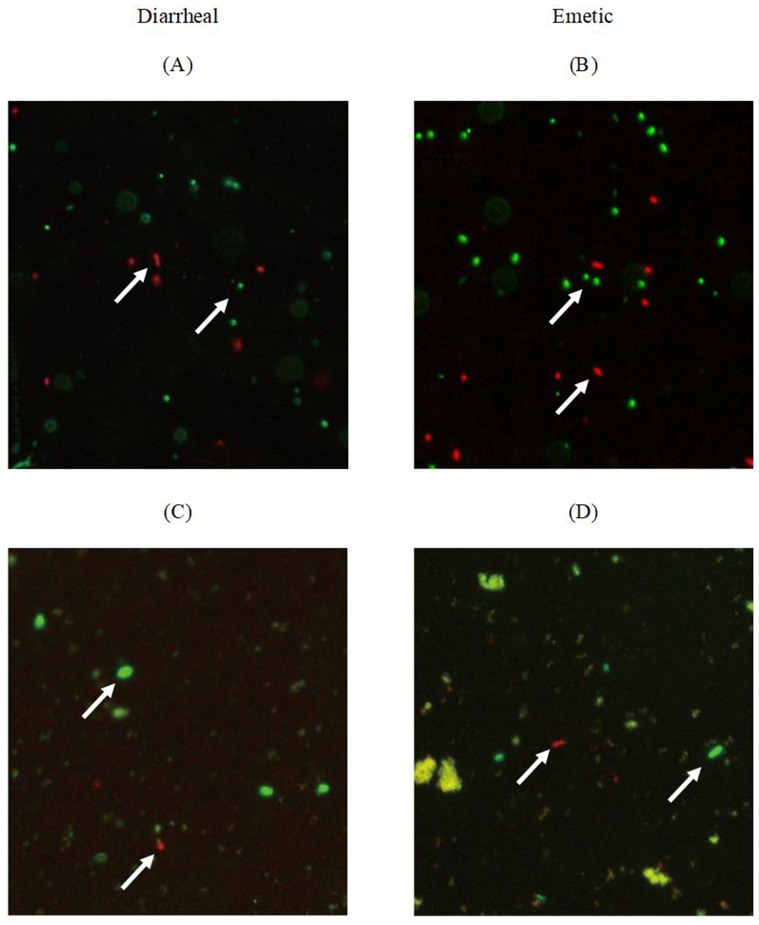
LIVE/DEAD BacLight staining of *B. cereus* biofilm cells on the stainless-steel surface and tofu (**A**) Diarrheal *B. cereus* on coupon; (**B**) Emetic *B. cereus* on coupon; (**C**) Diarrheal *B. cereus* on tofu; (**D**) Emetic *B. cereus* on tofu. White arrow indicates the live (green) or dead (red) cells.

The viability of biofilm cells on stainless-steel coupons and transferred cells to tofu was confirmed by fluorescence microscopy. Biofilm cells of diarrheal *B. cereus* (Figure 2A) and emetic *B. cereus* (Figure 2B) coexisted as live cells (fluorescent green) and dead cells (fluorescent red). Most cells were stained green, indicating live state when biofilm formed on stainless steel surfaces. Transferred diarrheal *B. cereus* (Figure 2C) and emetic *B. cereus* (Figure 2D) of biofilm cells to tofu also coexisted as live and dead cells, indicating possible regrowth of biofilm cells in tofu.

### 3.4. Growth of Transferred Biofilm Cells in Tofu

**Figure 3:**
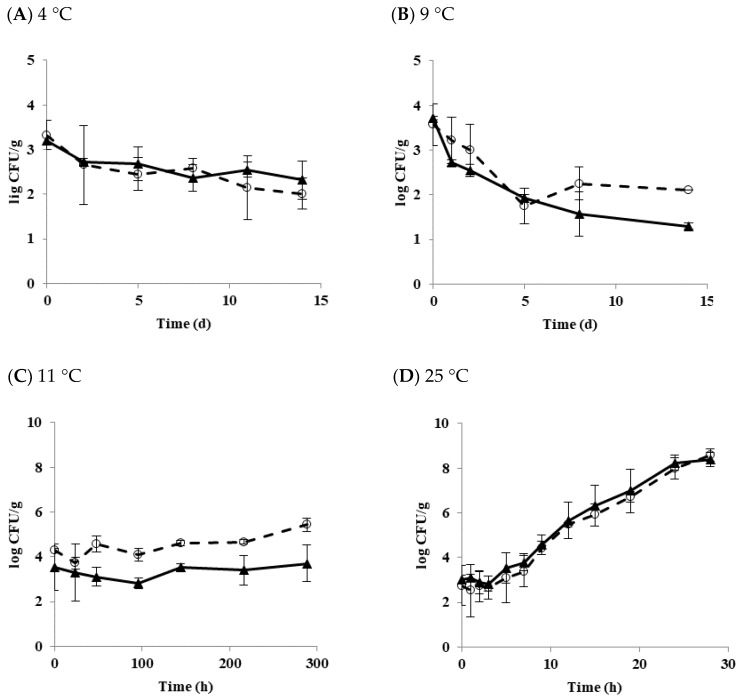
The growth and survival characteristics of transferred *B. cereus* biofilm cells in tofu as a function of temperature. (**A**) 4 °C; (**B**) 9 °C; (**C**) 11 °C; (**D**) 25 °C; ◯ Diarrheal strain; ▲ Emetic strain.

Matured biofilm cells may be transferred to form another biofilm on a new surface via planktonic growth [34]. Figure 3 shows the growth and survival characteristics of biofilm cells transferred to the tofu and stored at 4, 9, 11, and 25 °C.

At 4 °C, populations of diarrheal and emetic *B. cereus* decreased by 1.32 and 0.89 log CFU/g after 14 days of storage, respectively (Figure 3A). At 9 °C, populations of diarrheal and emetic *B. cereus* decreased by 1.46 and 2.42 log CFU/g, respectively (Figure 3B). These results indicate that populations of both types of *B. cereus* are more stable at 4 °C than 9 °C. Generally, biofilm cells are known to be more resistant to other environmental stresses than planktonic cells [35]. Multispecies strains are also more resistant to physical and chemical treatments when they form biofilms than a single strain [36]. Moreover, it was observed that the higher the density of bacteria, the more resistant to stress [37]. The initial concentration of biofilm cells transferred to tofu in the present study was low (in the range of 3–4 log CFU/g). Biofilm cells formed from a single diarrheal or emetic strain in this work did show resistance to low-temperature stress. Their populations also decreased at 4 and 9 °C, as shown in results with *B. cereus* planktonic cells in tofu. On the other hand, the population of diarrheal *B. cereus* biofilm cells increased by 1.16 log CFU/g at 11 °C for 12 days of storage, whereas the emetic *B. cereus* biofilm cells hardly grew, showing an increase of only 0.16 log CFU/g (Figure 3C). At 25 °C, both types of strains showed the rapid growth of about 8 log CFU/g after 28 h. LT and SGR values were 3.81 h and 0.33 log CFU/h for the diarrheal *B. cereus* and 3.48 h and 0.33 log CFU/h for the emetic *B. cereus*, respectively (Figure 3D). Results of the present study showed that transferred biofilm cells from the food-contact surface to tofu could grow under appropriate conditions. This result was similar to the results of Jeon et al. [15], who also found that the transferred biofilm cells to duck meat grew to maximum population density. Therefore, it is critical to prevent biofilm formation in the tofu manufacturing facility.

### 3.5. Effect of Temperature on Vegetable Cells and Spores of B. cereus and D Value in Tofu

**Figure 4:**
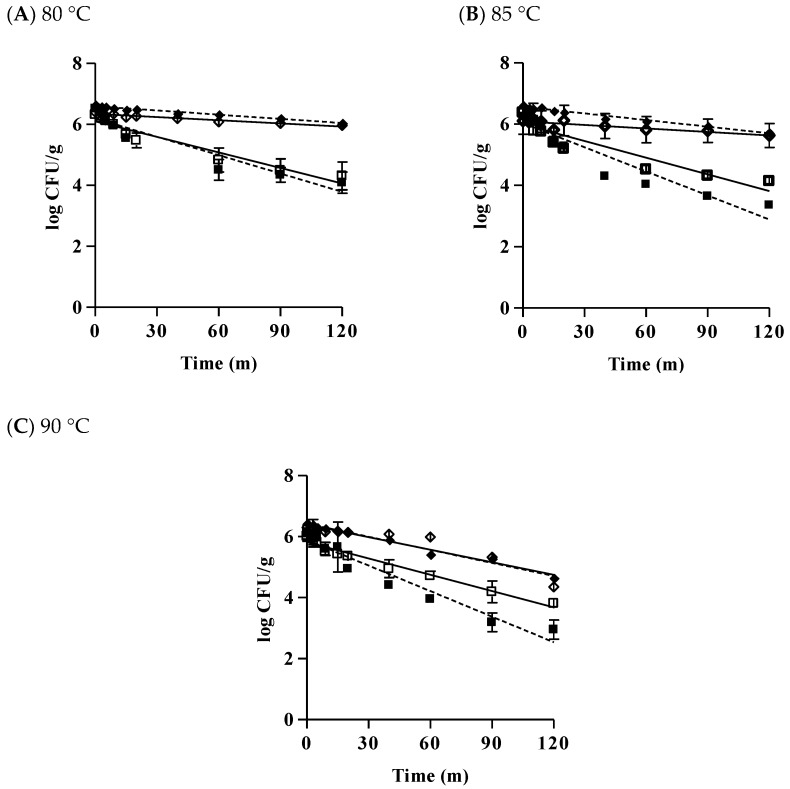
Time-dependent reduction in the *B. cereus* populations present in tofu heated at 80 °C (**A**), 85 °C (**B**), 90 °C (**C**). ■ Diarrheal vegetative cell; ◆ Diarrheal spore; □ Emetic vegetative cell; ◇ Emetic spore.

Figure 4 shows the effect of temperature on populations of *B. cereus* in tofu. At 80 °C, vegetative cells of both diarrheal and emetic *B. cereus* reduced by approximately 2 log CFU/g within 1 h whereas spores of diarrheal and emetic *B. cereus* only reduced by 0.60 log CFU/g and 0.44 log CFU/g, respectively, even after heating at 80 °C for 2 ho (Figure 4A).

At 85 °C, spores also survived better than vegetative cells after heat treatment for 2 ho. Vegetative cells of diarrheal *B. cereus* and emetic *B. cereus* reduced by 2.89 and 2.24 log CFU/g, respectively. However, diarrheal and emetic spores only reduced by 0.9 and 0.56 log CFU/g for the same time, respectively (Figure 4B). At 90 °C, more than 3 log CFU/g of vegetative cells of diarrheal *B. cereus* decreased while the emetic vegetative cells reduced by only 2.2 log CFU/g. On the other hand, diarrhea and emetic spores decreased by approximately 2 log CFU/g after heat treatment for 2 ho (Figure 4C). In this work, emetic spores showed the best survival ability, followed by diarrheal spores, emetic vegetative cells, and diarrheal vegetative cells. This result indicates that emetic *B. cereus* had better survival ability than diarrheal *B. cereus* during heat treatment. Spores were more resistant to heat treatment. This resistance is associated with dipicolinic acid (DPA) located in the spore core [38].

The results of this study were in agreement with previous studies [39]. Heating *B. cereus* vegetative cells in multi-grain soy milk to 65 °C for 1 min can significantly reduce the population of vegetative cells by 3.5–3.6 log CFU/g [23]. *B. cereus* spores in Kochujang decreased by 0.56 log CFU/g after heating for 15 min at 85 °C [40]. Various factors influence the difference in the survival ability of *B. cereus* in food. In general, the thermal resistance of bacteria is higher in solid food than in liquid food. The thermal resistance of *B. cereus* can be higher in tofu than in other foods. The composition of heating menstruum can also influence the thermal resistance of bacteria. Furthermore, high levels of carbohydrates, fat, protein, and minerals in menstruum could increase thermal resistance [41,42]. Although the specific nutritional content of each food is unknown, components of tofu might have produced these differences because it is generally known that tofu has high contents of carbohydrates, protein, and various minerals [43]. Differences in experimental strains [44], pH [45], aw [46], and numbers and densities of bacteria [47] might have also affected bacterial resistance to heat.

Table 3 shows the D-values of *B. cereus* in tofu after heating for 2 ho at 80, 85, and 90 °C. At 80 °C, emetic *B. cereus* spore had the highest D-value (288.30 min), followed by diarrheal *B. cereus* spore (218.30 min), emetic *B. cereus* vegetative cell (62.25 min), and diarrheal *B. cereus* vegetative cell (50.29 min). When heated at 85 °C, emetic spore also had the highest D-value (260.67 min), followed by diarrheal spore (139.83 min), emetic vegetative cell (40.96 min), and diarrheal vegetative cell (38.84 min). At 90 °C, no significant difference in D-value was observed between emetic and diarrheal spores. However, a significantly higher D-value of the emetic vegetative cell (56.43 min) than that of the diarrheal vegetative cell (36.91 min) was observed. In this work, D-values of diarrheal and emetic spores significantly decreased when the temperature was increased (*p* < 0.05). However, no significant differences in D-value of vegetative cells between these two types of strains were observed when the temperature was increased (*p* > 0.05). At all temperatures, the D-value of spores was higher than that of vegetative cells. The emetic *B. cereus* used in this study was more resistant to heating than the diarrheal *B. cereus*.

The D-value for *B. cereus* spores in pork luncheon roll is 29.5 min at 85 °C and 10.1 min at 90 °C [39]. Cho et al. [48] investigated the D-value of diarrheal *B. cereus* spores in Sunshik using the same strain (ATCC 11778) as used in this work and found that the D-value of spores in Sunshik is 22.52 min at 80 °C, 4.91 min at 85 °C, and 3.08 min at 90 °C. D-values of *B. cereus* spores in tofu were higher than those of previous studies [39,48], indicating that *B. cereus* spores in tofu are more resistant than those in pork luncheon roll and Sunshik. D-values of vegetative cells in tofu in this work were also higher than those in other studies [39,49]. D-values of *B. cereus* vegetative cells were 1.0–33.2 min after heat treatment at 50–60 °C in pork luncheon roll [39]. D-values of vegetative cells ranged from 3.55 min at 60 °C to 7.65 min at 56 °C in whole milk [49]. Packaged tofu is generally recommended to be sterilized at 87 ± 2 °C for 32 ± 2 min to ensure microbiological safety [50]. Results of the present study showed that vegetative cells and spores of *B. cereus* contaminated in tofu at high concentrations (>6 logs CFU/g) were not completely killed by heating for 2 ho, regardless of heating temperature. Therefore, the initial contamination of *B. cereus* in tofu must be controlled. Furthermore, proper sterilization temperature and time must be applied to reduce bacteria cells to a safe range without affecting the physical properties or taste of tofu.

## 4. Conclusions

*Bacillus cereus* is an endospore-forming pathogen that can cause a diarrheal and emetic type of food poisoning. In this work, we observed that both diarrheal and emetic *B. cereus* do not grow in tofu at a temperature below 9 °C. Overall, populations of both diarrheal and emetic *B. cereus* were more stable at 4 °C than 9 °C. The emetic *B. cereus* showed higher risk than the diarrheal *B. cereus* due to better survival ability at 4 °C and faster growth at a temperature above 11 °C. The populations of both diarrheal and emetic *B. cereus* in tofu did not change at 10 °C. Transferred biofilm cells to tofu coexisted as live and dead cells and behaved like planktonic cells in tofu. Tofu is often used as the main ingredient in salads and consumed as a ready-to-eat food. Therefore, effective cleaning and sanitizing to control biofilm formation should be carefully managed in a tofu manufacturing environment along with temperature control at the retail market for the safe consumption of tofu.

## Figures and Tables

**Table 1:** The growth and survival kinetic parameters of *B. cereus* planktonic cells in tofu.

Strain	Storage Temperature (°C)	Delta (d)	*p*	LT(h)	SGR (logCFU/h)
**Diarrheal**	4	5.15 ± 1.08 *	0.98 ± 0.02	-	-
**Emetic**		14.00 ± 2.69	0.43 ± 0.02		
**Diarrheal**	9	12.88 ± 3.31 *	0.88 ± 0.14	-	-
**Emetic**		22.59 ± 5.25	0.44 ± 0.05		
**Diarrheal**	11	-	-	79.67 ± 8.43 *	0.02 ± 0.00
**Emetic**				45.39 ± 8.87	0.02 ± 0.00
**Diarrheal**	25	-	-	9.53 ± 0.94 *	0.31 ± 0.02 *
**Emetic**				4.34 ± 0.21	0.55 ± 0.04

* indicates statistically significant differences between diarrheal and emetic strains by *t*-test (*p* < 0.05).

**Table 2:** Biofilm formation of *B. cereus* on stainless steel and efficiency of transfer (EOT) from biofilm cells to tofu.

Strain	Biofilm Formation (Log CFU/Coupon)	Efficiency of Transfer (EOT)
**Diarrheal**	4.01 ± 0.51	0.72 ± 0.05
**Emetic**	4.00 ± 0.71	0.81 ± 0.12

**Table 3:** D-values of diarrheal and emetic *B. cereus* strain in tofu.

Strain Type		D-Values (min)		
		80 °C	85 °C	90 °C
**Diarrheal**	**spore**	^A^ 218.30 ± 6.36 ^b^	^B^ 139.83 ± 10.61 ^b^	^C^ 70.78 ± 6.25 ^a^
	**vegetative cell**	^A^ 50.29 ± 6.24 ^c^	^A^ 38.84 ± 0.19 ^d^	^A^ 36.91 ± 4.39 ^c^
**Emetic**	**spore**	^A^ 288.30 ± 14.28 ^a^	^B^ 260.67 ± 4.40 ^a^	^C^ 70.12 ± 2.47 ^a^
	**vegetative cell**	^A^ 62.25 ± 17.11 ^c^	^A^ 56.52 ± 0.06 ^c^	^A^ 56.43 ± 5.49 ^b^

^A–C^ Mean values (*n* = 3) in the same row with different letters are significantly different (*p* <0.05), ^a–d^ Mean values (*n* = 3) in the same column with different letters are significantly different (*p* <0.05).

## References

[1] Préstamo G., Fontecha J. (2007). High pressure treatment on the tofu fatty acids and acylglycerols content. Innov. Food Sci. Emerg. Technol..

[2] Shin I.S., Han J.S., Choi K.D., Chung D.H., Choi G.P., Ahn J.H. (2010). Effect of isothiocyanates from horseradish (Armoracia rusticana) on the quality and shelf life of tofu. Food Control..

[3] Ananchaipattana C., Hosotani Y., Kawasaki S., Pongswat S., Latiful B.M., Isobe S., Inatsu Y. (2012). Bacterial contamination of soybean curd (tofu) sold in Thailand. Food Sci. Technol. Res..

[4] Lee D.Y., Kwon K.H., Chai C.H., Oh S.W. (2017). Microbial contamination of tofu in Korea and growth characteristics of Bacillus cereus isolates in Tofu. Lwt-Food Sci. Technol..

[5] Sonenshein A.L., Hoch J.A., Losick R. (2002). Bacillus Subtilis and Its Closest Relatives.

[6] Setlow P. (2006). Spores of Bacillus subtilis: Their resistance to and killing by radiation, heat and chemicals. J. Appl. Microbiol..

[7] Sarrıas J.A., Valero M., Salmeron M.C. (2003). Elimination of Bacillus cereus contamination in raw rice by electron beam irradiation. Food Microbiol..

[8] Hood S.K., Zottola E.A. (1995). Biofilms in food processing. Food Control..

[9] Lee S.H., Park Y.S. (2012). Analysis of Microflora Profile in the Manufacturing Process of Commercial Tofu. Food Eng. Prog..

[10] Rossi F., Felis G.E., Martinelli A., Calcavecchia B., Torriani S. (2016). Microbiological characteristics of fresh tofu produced in small industrial scale and identification of specific spoiling microorganisms (SSO). Lwt-Food Sci. Technol..

[11] Oh K.Y., Ahn S.C., Oh S.M. (2016). A study of shelf-life and antimicrobial activity on putrefactive microorganisms related to soybean curd of Persicaria hydropiper L. extracts. Culin. Sci. Hosp. Res..

[12] Park K.N., Park L.Y., Kim D.G., Park G.S., Lee S.H. (2007). Effect of turmeric (Curcuma aromatica Salab.) on shelf life of tofu. Korean J. Food Preserv..

[13] Gibson A.M., Bratchell N., Roberts T.A. (1987). The effect of sodium chloride and temperature on the rate and extent of growth of Clostridium botulinum type A in pasteurized pork slurry. J. Appl. Bacteriol..

[14] Geeraerd A.H., Valdramidis V.P., Van Impe J.F. (2005). GInaFiT, a freeware tool to assess non-log-linear microbial survivor curves. Int. J. Food Microbiol..

[15] Jeon H.R., Kwon M.J., Yoon K.S. (2018). Control of Listeria innocua biofilms on food contact surfaces with slightly acidic electrolyzed water and the risk of biofilm cells transfer to duck meat. J. Food Prot..

[16] Antoniou K., Frank J.F. (2005). Removal of Pseudomonas putida biofilm and associated extracellular polymeric substances from stainless steel by alkali cleaning. J. Food Prot..

[17] Sadekuzzaman M., Yang S., Mizan M.F.R., Kim H.S., Ha S.D. (2017). Effectiveness of a phage cocktail as a biocontrol agent against L. monocytogenes biofilms. Food Control..

[18] Kreske A.C., Ryu J.H., Pettigrew C.A., Beuchat L.R. (2006). Lethality of chlorine, chlorine dioxide, and a commercial produce sanitizer to Bacillus cereus and Pseudomonas in a liquid detergent, on stainless steel, and in biofilm. J. Food Prot..

[19] Midelet G., Kobilinsky A., Carpentier B. (2006). Construction and analysis of fractional multifactorial designs to study attachment strength and transfer of Listeria monocytogenes from pure or mixed biofilms after contact with a solid model food. Appl. Environ. Microbiol..

[20] Montville R., Chen Y., Schaffner D.W. (2001). Glove barriers to bacterial cross-contamination between hands to food. J. Food Prot..

[21] Wang W., Tao R., Tong Z., Ding Y., Kuang R., Zhai S., Liu J., Ni L. (2012). Effect of a novel antimicrobial peptide chrysophsin-1 on oral pathogens and Streptococcus mutans biofilms. Peptides.

[22] Mazas M., Gonzalez I., Lopez M., González J., Sarmiento R.M. (1995). Effects of sporulation media and strain on thermal resistance of Bacillus cereus spores. Int. J. Food Sci. Tech..

[23] Kim N.H., Koo J.M., Rhee M.S. (2015). Thermal resistance characteristics of bacillus cereus, Escherichia coli O157: H7, and listeria monocytogenes in a multi-grain soy milk product. Korean J. Food Nutr..

[24] Albert I., Mafart P. (2005). A modified Weibull model for bacterial inactivation. Int. J. Food Microbiol..

[25] MFDS Food Code, Preservation and Distribution Standards. http://www.foodsafetykorea.go.kr/foodcode/01_03.jsp?idx=13.

[26] Kang K.A., Kim Y.W., Yoon K.S. (2010). Development of predictive growth models for Staphylococcus aureus and Bacillus cereus on various food matrices consisting of ready-to-eat (RTE) foods. Food Sci. Anim. Resour..

[27] Liu J.G., Lin T.S., Lin W.Y. (2010). Evaluating the growth of Listeria monocytogenes that has been inoculated into tofu containing background microflora. Food Control..

[28] Auger S., Ramarao N., Faille C., Fouet A., Aymerich S., Gohar M. (2009). Biofilm formation and cell surface properties among pathogenic and nonpathogenic strains of the Bacillus cereus group. Appl. Environ. Microbiol..

[29] Oliveira R., Melo L.F., Oliveira A., Salgueiro R. (1994). Polysaccharide production and biofilm formation by Pseudomonas fluorescens: Effects of pH and surface material. Colloids Surf. B Biointerfaces.

[30] Pagedar A., Singh J., Batish V.K. (2010). Surface hydrophobicity, nutritional contents affect Staphylococcus aureus biofilms and temperature influences its survival in preformed biofilms. J. Basic Microbiol..

[31] Garrett T.R., Bhakoo M., Zhang Z. (2008). Bacterial adhesion and biofilms on surfaces. Prog. Nat. Sci..

[32] Jensen D.A., Friedrich L.M., Harris L.J., Danyluk M.D., Schaffner D.W. (2013). Quantifying transfer rates of Salmonella and Escherichia coli O157: H7 between fresh-cut produce and common kitchen surfaces. J. Food Prot..

[33] Rodriguez A., McLandsborough L.A. (2007). Evaluation of the transfer of Listeria monocytogenes from stainless steel and high-density polyethylene to Bologna and American cheese. J. Food Prot..

[34] Toyofuku M., Inaba T., Kiyokawa T., Obana N., Yawata Y., Nomura N. (2016). Environmental factors that shape biofilm formation. Biosci. Biotechnol. Biochem..

[35] Smith J., Fratamico P.M., Uhlich G. (2009). Molecular mechanisms involved in biofilm formation by food-associated bacteria. Biofilms in the Food and Beverage Industries.

[36] Simoes M., Simoes L.C., Vieira M.J. (2009). Species association increases biofilm resistance to chemical and mechanical treatments. Water Res..

[37] Hingston P.A., Stea E.C., Knøchel S., Hansen T. (2013). Role of initial contamination levels, biofilm maturity and presence of salt and fat on desiccation survival of Listeria monocytogenes on stainless steel surfaces. Food Microbiol..

[38] Baweja R.B., Zaman M.S., Mattoo A.R., Sharma K., Tripathi V., Aggarwal A., Dubey G.P., Kurupati R.K., Ganguli M., Chaudhury N.K. (2008). Properties of Bacillus anthracis spores prepared under various environmental conditions. Arch. Microbiol..

[39] Byrne B., Dunne G., Bolton D.J. (2006). Thermal inactivation of Bacillus cereus and Clostridium perfringens vegetative cells and spores in pork luncheon roll. Food Microbiol..

[40] Kim Y.S., Ahn Y.S., Jeong D.Y., Shin D.H. (2008). Isolation and identification of Bacillus cereus from fermented red pepper-soybean paste (kochujang), and its heat resistance characteristics. Food Sci. Biotechnol..

[41] Marquis R.E., Shin S.Y. (1994). Mineralization and responses of bacterial spores to heat and oxidative agents. Fems Microbiol. Rev..

[42] Oteiza J.M., Giannuzzi L., Califano A.N. (2003). Thermal inactivation of Escherichia coli O157: H7 and Escherichia coli isolated from morcilla as affected by composition of the product. Food Res. Int..

[43] Kim G.W., Kim G.H., Kim J.S., An H.Y., Hu G.W., Son J.K., Kim O.S., Cho S.Y. (2008). Quality of tofu prepared with deep seawater as coagulant. Korean J. Fish. Aquat. Sci..

[44] Choma C., Guinebretiere M.H., Carlin F., Schmitt P., Velge P., Granum P.E., Nguyen-The C. (2000). Prevalence, characterization and growth of Bacillus cereus in commercial cooked chilled foods containing vegetables. J. Appl. Microbiol..

[45] Casadei M.A., Ingram R., Hitchings E., Archer J., Gaze J.E. (2001). Heat resistance of Bacillus cereus, Salmonella typhimurium and Lactobacillus delbrueckii in relation to pH and ethanol. Int. J. Food Microbiol..

[46] Gaillard S., Leguérinel I., Mafart P. (1998). Model for combined effects of temperature, pH and water activity on thermal inactivation of Bacillus cereus spores. J. Food Sci..

[47] Humpheson L., Adams M.R., Anderson W.A., Cole M.B. (1998). Biphasic thermal inactivation kinetics in Salmonella enteritidis PT4. Appl. Environ. Microbiol..

[48] Cho Y.S., Jung E.Y., Lee M.K., Yang C.Y., Shin D.B. (2008). Survival, isolation and characterization of Bacillus cereus from Sunshik. J. Food Hyg. Saf..

[49] Desai S.V., Varadaraj M.C. (2010). Behavioural pattern of vegetative cells and spores of Bacillus cereus as affected by time-temperature combinations used in processing of Indian traditional foods. J. Food Sci. Technol..

[50] MFDS Tofu HACCP. http://www.mfds.go.kr/index.do?mid=1486&seq=11933&cmd=v.

